# Characterization of a complex context containing *mecA* but lacking genes encoding cassette chromosome recombinases in *Staphylococcus haemolyticus*

**DOI:** 10.1186/1471-2180-13-64

**Published:** 2013-03-22

**Authors:** Zhiyong Zong

**Affiliations:** 1 Center of Infectious Diseases, West China Hospital, Sichuan University, Guoxuexiang 37, Chengdu, 610041, China; 2 State Key Laboratory of Biotherapy, Sichuan University, Chengdu, China

## Abstract

**Background:**

Methicillin resistance determinant *mecA* is generally transferred by SCC*mec* elements. However, the *mecA* gene might not be carried by a SCC*mec* in a *Staphylococcus haemolyticus* clinical isolate, WCH1, as no cassette chromosome recombinase genes were detected. Therefore, the genetic context of *mecA* in WCH1 was investigated.

**Results:**

A 40-kb region containing *mecA* was obtained from WCH1, bounded by orfX at one end and several orfs of *S. haemolyticus* core chromosome at the other. This 40-kb region was very complex in structure with multiple genetic components that appeared to have different origins. For instance, the 3.7-kb structure adjacent to orfX was almost identical to that on the chromosome of *Staphylococcus epidermidis* RP62a but was absent from *S. haemolyticus* JCSC1435. Terminal inverted repeats of SCC were found but no *ccr* genes could be detected. *mecA* was bracketed by two copies of IS*431*, which was flanked by 8-bp direct target repeat sequence (DR).

**Conclusions:**

The presence of 8-bp DR suggests that the two copies of IS*431* might have formed a composite transposon for mobilizing *mecA*. This finding is of significance as multiple copies of IS*431* are commonly present in the contexts of *mecA*, which might have the potential to form various composite transposons that could mediate the mobilization of *mecA*. This study also provides an explanation for the absence of *ccr* in some staphylococci isolates carrying *mecA*.

## Background

Methicillin-resistant staphylococci represent a great challenge for treatment and public health. In staphylococci, methicillin resistance is mainly due to the expression of the *mecA* gene, which specifies penicillin binding protein 2a (PBP2a), a transpeptidase with a low affinity for β-lactams [1,2]. *mecA* is carried by a mobile genetic element (MGE) termed the staphylococcal cassette chromosome *mec* (SCC*mec*) [2,3]. Generally, SCC*mec* contains two essential components, i.e. the *mec* gene complex and the *ccr* gene complex. The *mec* gene complex consists of *mecA*, the regulatory genes and associated insertion sequences and has been classified into six different classes, i.e. A, B, C1, C2, D and E. Cassette chromosome recombinase (*ccr*) genes (*ccrC* or the pair of *ccrA* and *ccrB*) encode recombinases mediating integration and excision of SCC*mec* into and from the chromosome [2,3]. The *ccr* gene(s) and surrounding genes form the *ccr* gene complex.

A *Staphylococcus haemolyticus* clinical isolate, WCH1, was found carrying *mecA* but no *ccr* genes. Although clinical isolates of *S. haemolyticus* containing *mecA* but lacking *ccr* genes have been reported previously [4-6], information about the detailed contexts of *mecA* is largely absent. The genetic context of *mecA* in WCH1 was therefore investigated using long-range PCR, PCR mapping, inverse PCR and sequencing as described previously [7].

## Results and discussion

**Figure 1:**
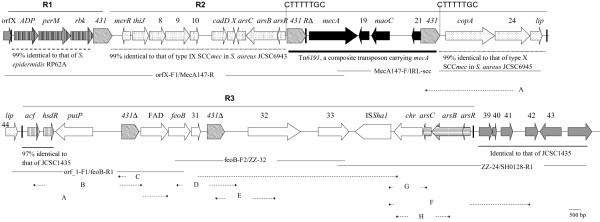
**The complex genetic context of *mecA *in WCH1.** The context of *mecA* is displayed in two parts with the same *lip* gene shown in both parts. Numbers of orf are shown (e.g. 8 represents orf8), while IS*431* is indicated as *431*. PCR primers for mapping and linking are indicated. JCSC1435 is a *S. haemolyticus* strain. Several self-ligated restricted fragments that were used as templates for inverse PCR were indicated as fragments **A** to **H** with the restriction locations of the enzymes being shown. The restriction enzymes and primers for inverse PCR for each fragment are as below: **A**. *Hind*III, orf2_1-R1/ZZ-4; **B**. *Hae*III, acf-R1/ZZ-3; **C**. *Nhe*I, orf24-1/ZZ-16; **D**. *Hha*I, feoB-F1/feoB-R1; **E**. *EcoR*I, ZZ-11/ZZ-12; **F**. *Hinc*II, ZZ-28/arsR-up1; **G**. *Hha*I, ZZ-28/ZZ-29; **H**. *EcoR*V, ZZ-30/ZZ-31. The 8-bp DR (CTTTTTGC) possibly generated by the insertion of Tn*6191* is indicated. Black poles represent the IR of SCC. Genes with different origins are shown in different shading with those belonging to the *mec* complex in black and those of the core chromosome of *S. haemolyticus* in grey. Closest matches, if available, of certain regions are indicated. More information on genes for their closet matches and function is available in Table 1.

The minimum inhibitory concentration (MIC) of cefoxitin against WCH1 was 128 μg/ml. A 40-kb region containing *mecA* was obtained from WCH1, abutting orfX at one end and seven orfs designated orf39 to orf44 here (Table 1), which were also present in the *oriC* environ on the chromosome of the completely-sequenced *S. haemolyticus* strain JCSC1435 (GenBank accession no. AP006716) [8], at the other (Figure 1). The partial sequence of orfX obtained was 99% identical to that of *S. haemolyticus* JCSC1435. orf39 to orf44 were identical to the counterparts of *S. haemolyticus* JCSC1435 and were not part of any known mobile genetic elements (MGE), confirming that these orfs indeed belonged to the core chromosome of *S. haemolyticus*.

**Table 1:** **Genes and MGE in the genetic context of *mecA *in WCH1**

**Gene or MGE**	**Position **^***a***^	**Product**	**Closest match **^***b,c***^
			**Identity, species strain**
orfX	1-316	Hypothetical protein	99%, *S. haemolyticus* JCSC1435
*ADP*	445-1431	ADP-ribosylglycohydrolase	99%, *S. epidermidis* RP62a (locus SERP2218)
*perM*	1450-2784	Cytosine/purines, uracil, thiamine, allantoin permease family protein	99%, *S. epidermidis* RP62a (locus SERP2217)
*rbk*Δ^d^	2781-3719	Ribokinase	99%, *S. epidermidis* RP62a (locus SERP2216)
IS*431*	3701-4401	IS*431*	
*merR*	4888-5235	Transcriptional regulator of the *merR* family	100%, type IX SCC*mec* of *S. aureus* JCSC6943 and *S. haemolyticus* JCSC1435 (locus SH0094)
*thiJ*	5313-5996	ThiJ/PfpI family protein	100%, type IX SCC*mec* of *S. aureus* JCSC6943 and *S. haemolyticus* JCSC1435 (locus SH0095)
orf8	6018-6683	NAD dependent epimerase/dehydratase family protein	100%, type IX SCC*mec* of *S. aureus* JCSC6943, type X SCC*mec* of *S. aureus* JCSC6945 and *S. haemolyticus* JCSC1435 (locus SH0095)
orf9	6687-7691	Oxidoreductase, zinc-binding dehydrogenase family protein	100%, type IX SCC*mec* of *S. aureus* JCSC6943 and *S. haemolyticus* JCSC1435 (locus SH0097)
orf10	7700-8065	Hypothetical protein of the COG4270 superfamily, predicted membrane protein	100%, type IX SCC*mec* of *S. aureus* JCSC6943, type X SCC*mec* of *S. aureus* JCSC6945 and *S. haemolyticus* JCSC1435 (locus SH0098)
*cadD*	8601-9218	Cadmium binding protein	100%, type IX SCC*mec* of *S. aureus* JCSC6943 and *S. haemolyticus* JCSC1435 (locus SH0099)
*cadX*	9237-9578	Cadmium resistant accessory protein	100%, type IX SCC*mec* of *S. aureus* JCSC6943 and *S. haemolyticus* JCSC1435 (locus SH0100)
*arsC*	9999-9598	Arsenate reductase	100%, type IX SCC*mec* of *S. aureus* JCSC6943 and *S. haemolyticus* JCSC1435 (locus SH0101)
*arsB*	11306-10017	Arsenical pump membrane protein	99%, type IX SCC*mec* of *S. aureus* JCSC6943 and *S. haemolyticus* JCSC1435 (locus SH0102)
*arsR*	11623-11302	Arsenical resistance operon repressor	100%, type IX SCC*mec* of *S. aureus* JCSC6943, type X SCC*mec* of *S. aureus* JCSC6945 and *S. haemolyticus* JCSC1435 (locus SH0103)
IS*431*	11697-12486	IS*431*	
*mecR*Δ	12503-12487	Signal transducer protein	
*mecA*	12603-14609	Penicillin binding protein 2a	
orf19	15083-14655	Hypothetical protein	
*maoC*	15923-15180	Putative acyl dehydratase maoc	
orf21	17208-16840	Putative HMG-CoA synthase (partial)	
IS*431*	17209-17998	IS*431*	
*copA*	18241-20262	Copper-transporting atpase	99%, type X SCC*mec* of *S. aureus* JCSC6945.
orf24	20277-21710	Putative multicopper oxidases	99%, *S. haemolyticus* JCSC1435 (locus SH0106)
*lip*	21730-22212	Lipoprotein	99%, *S. aureus* JCSC6943
*acf*	22588-23073	Putative Acyl-CoA acyltransferase	97%, *S. haemolyticus* JCSC1435 (locus SH0117)
*hsdR*	23254-23667	Type I restriction endonuclease, HsdR	97%, *S. haemolyticus* JCSC1435(locus SH0118)
*putP*	25274-23736	Sodium/proline symporter (High affinity proline permease)	78%, *S. saprophyticus* ATCC 15305 (locus SSP0399)
IS*431*Δ	26462-27184	IS*431*, truncated	
*FAD*	27261-28382	FAD-dependent pyridine nucleotide-disulphide oxidoreductase	66%, a few *S. aureus* strains, e.g. COL
*feoB*	28376-29272	FeoB family ferrous iron transporter	68% (partially, from position 28804 to 29216), *S. carnosus* TM300
orf31	29337-29717	Putative transmembrane protein	73% (partially, from position 29438 to 29618), *S. aureus* MSHR1132
IS*431*Δ^e^	30690-29891	IS*431*, incomplete due to internal termination	
orf32	31660-33822	ABC-type bacteriocin transporter family protein	71%, *S. epidermidis* plasmid SAP105A
orf33	34541-35809	DUF867 type protein, putative phage-related protein	71% (partially from position 35252), *S. epidermidis* ATCC 12228
IS*Sha1*	37543-36061	IS*Sha1*	98%, *S. haemolyticus* JCSC1435
*chr*	38832-37669	Chromate transporter	66% (partially from position 37895 to 38782), *Oceanobacillus iheyensis* HTE831
*arsC*	39261-38869	Arsenate reductase	97%, *S. aureus* strains LGA251 and M10/0061
*arsB*	40577-39279	Arsenical pump membrane protein	92%, *S. xylosus* plasmid pSX267
*arsR*	40885-40571	Arsenical resistance operon repressor	91%, *S. aureus* plasmid SAP099B and EDINA
orf39	41223-41771	DUF576 type protein	100%, *S. haemolyticus* JCSC1435 (locus SH0120)
orf40	41768-41935	Hypothetical protein	100%, *S. haemolyticus* JCSC1435 (locus SH0121)
orf41	42126-43013	Hypothetical protein, similar to mechanosensitive ion channel Mscs, transmembrane protein	100%, *S. haemolyticus* JCSC1435 (locus SH0122)
orf42	43522-44046	DUF3267 type protein	100%, *S. haemolyticus* JCSC1435 (locus SH0123)
orf43	44998-44120	Hypothetical protein, similar to cobalamin synthesis related protein CobW	100%, *S. haemolyticus* JCSC1435 (locus SH0124)
orf44	45625-46248	Hypothetical protein, similar to Zn-binding lipoprotein AdcA	100%, *S. haemolyticus* JCSC1435 (locus SH0125)

^*a*^ Positions are according to GenBank accession no. JQ764731.

^*b*^ GenBank accession no.: *S. aureus* LGA251 (FR821779), *S. aureus* JCSC6943 (AB505628), *S. aureus* JCSC6945 (AB505630), *S. aureus* M10/0061 (FR823292), *S. aureus* MSHR1132 (FR821777), *S. carnosus* TM300 (AM295250), *S. epidermidis* ATCC 12228 (AE015929), *S. epidermidis* RP62a (CP000029), *S. haemolyticus* JCSC1435 (AP006716), *S. saprophyticus* ATCC 15305 (AP008934), *Oceanobacillus iheyensis* HTE831 (BA000028), *S. aureus* plasmid SAP099B (GQ900449), *S. aureus* plasmid EDINA (AP003089), *S. epidermidis* plasmid SAP105A (GQ900452), *S. xylosus* plasmid pSX267 (M80565).

^*c*^ Closest matches of MGE (IS*431* and IS*Sha1*) and genes belonging to the *mec* complex are not listed as there are many identical matches.

^*d*^ Truncated by IS*431* with 19 bp of the 3′ end missing and the read frame extending into IS*431*.

^*e*^ The *tnpA* of IS*431* was terminated prematurely due to internal point mutation.

### *mecA* is bracketed by two copies of IS*431* flanking by an 8-bp direct repeat sequence

WCH1 had a class C1 *mec* gene complex composed of *mecA*, *mecR1Δ* truncated by the insertion of the insertion sequence IS*431*, several other genes and another copy of IS*431* downstream of *mecA* with the two copies of IS*431* at the same orientation (Figure 1)*.* The class C1 *mec* gene complex is also present in SCC*mec* types VII and X of *Staphylococcus aureus* and several unnamed types of SCC*mec* in coagulase-negative staphylococci (CoNS) [9].

An 8-bp identical sequence (CTTTTTGC; Figure 1) was identified flanking the two copies of IS*431*. The 8-bp DR was part of the spacer sequence between *arsR* (encoding an arsenical resistance operon repressor) and *copA* (encoding a copper-exporting ATPase). The presence of a direct repeat (DR) suggested that the two copies of IS*431* might have formed a composite transposon with the potential to mediate the mobilization of *mecA* into different genomic locations. This *mecA*-carrying IS*431*-formed composite transposon was designated Tn*6191* according to the transposon database (http://www.ucl.ac.uk/eastman/tn/). Composite transposons formed by IS*431* generating 8-bp AT-rich DR on insertion have been seen before, such as Tn*6072* carrying *ccrC* and the aminoglycoside resistance determinant *aacA* found in a ST239 *S. aureus*[10]. Two copies of IS*431* have also been found to mediate the transposition of plasmids pUB110 encoding bleomycin resistance [11] and pT181 encoding tetracycline and mercury resistance [12]. However, Tn*6072* and other IS*431*-formed composite transposons do not contain *mecA*. IS*431* is widely distributed in *Staphylococcus epidermidis*, *S. haemolyticus* and methicillin-resistant *S. aureus* (MRSA) [13] and appears to play a vital role in generating mosaicism in the genetic contexts of *mecA*. The insertion of IS*431* and homologous recombination between different copies of IS*431* can result in acquisition, loss and re-arrangements of genetic components [14,15]. Therefore, IS*431* apparently serves as the “adapters” allowing genetic components to be linked and clustered together to form complicated genetic contexts of *mecA*.

In GenBank and literature, e.g. [3], there are many cases in which *mecA* is bracketed by two copies of IS*431*, either at the same or opposite orientations, i.e. the class C1 or C2 *mec* complex. In these cases, two copies of IS*431* have the potential to form a composite transposon mediating the mobilization of *mecA* but no 8-bp DR could be identified flanking the class C1 or C2 *mec* complexes. This suggests that the two copies of IS*431* might have inserted in tandem rather than mobilize together as a unit. Alternatively, IS*431* might behave likes IS*26*[16], an insertion sequence also of the IS*6* family, that could lead to adjacent deletions, leaving no DR.

No *ccr* genes could be identified in this large region containing *mecA*. In the 1970s and 1980s, it was found that methicillin resistance could be transferred by phages [17-21] in experimental conditions and could be also carried by a transposon, Tn*4291*, located on a naturally occurring plasmid, pI524 [21]. However, these studies were carried out before the identification of *mecA* and no sequence information was available for the phages carrying methicillin resistance, Tn*4291* and pI524. It remains unclear whether methicillin resistance in these experiments was due to the expression of *mecA*. In particular, Tn*4291* mediated resistance to methicillin but not to penicillin, raising the possibility that the methicillin resistance determinant carried by Tn*4291* was actually not *mecA*. *mecA* is usually transferred by SCC*mec*, but *mecA* existed in the absence of any known types of *ccr* genes have been found in both MRSA and CoNS previously. In particular, no known *ccr* genes were detected for an half of methicillin-resistant *S. haemolyticus* isolates from a hospital in Tunisia [22], suggesting that elements carrying *mecA* but lacking *ccr* genes might be common in *S. haemolyticus*. However, the detailed genetic context of *mecA* were not characterized in these cases and therefore the exact reasons for the absence of *ccr* genes remain unclear [2]. The present study provides a detailed example that *mecA* was in a context without *ccr* genes and might be able to be transferred by a MGE other than SCC*mec*.

### A complex SCC-like remnant containing components with various origins

This 40-kb region between orfX and orf39 contained five copies of IS*431* (designated IS*431*-1 to −5 from upstream of to downstream of *mecA*, respectively) and three terminal inverted repeats (IR) of SCC elements (Figure 1). An IR was in the orfX, designated IR1 here. The second IR another was located between the lipoprotein-encoding gene, *lip*, and a putative Acyl-CoA acyltransferase-encoding gene, *acf*, designated IR2 here. The third IR was adjacent to orf39, part of the core chromosome of *S. haemolyticus*, designated IR3 here (Figure 1). This 40-kb region was actually bracketed by two IR, IR1 and IR3, resembling the remnant of a SCC-like element but without *ccr* genes. In light of the presence of an internal IR, IR2, this *ccr*-absent large region was a remnant of a composite SCC element or comprised remnants of multiple SCC elements.

The 3.7-kb region between orfX and the IS*431*-1 was designated R1 (representing region 1) and contained genes encoding ADP-ribosylglycohydrolase, permease and ribokinase. R1 was almost identical to the counterpart (loci SERP2216 to SERP2218) of the integrative plasmid *v*Se1 on the chromosome of *S. epidermidis* RP62a (GenBank accession no. CP000029) but was absent from *S. haemolyticus* JCSC1435, suggesting a foreign origin. Of note, the ribokinase-encoding gene, *rbk*, was truncated at the 3′ end by the insertion of IS*431*, leaving a 920 bp remnant of the 939 bp gene.

The region between the IS*431*-1 and IR2 was designated R2. As mentioned above, Tn*6191* was inserted into the spacer between *arsR* and *copA* in R2. Besides Tn*6191*, R2 also contained a few genes, the *cadXD* operon mediating resistance to cadmium and the *ars* operon required for detoxifying arsenate. In R2, the sequence from the IS*431*-1 to *arsB* was closest (99.9% similarity) to the counterpart in the type IX SCC*mec* of *S. aureus* strain JCSC6943 (GenBank accession no. AB505628), while that from *arsB* to IR2 excluding Tn*6191* was almost identical to the corresponding region in the type X SCC*mec* of *S. aureus* JCSC6945 (GenBank accession no. AB505630). This suggests that R2 might have resulted from homologous recombination between the *ars* operons of the type IX and X SCC*mec*. R1 and R2 had different origins and were separated by a single copy of IS*431*, suggesting that IS*431* served as a joining point that brought the two regions together.

The large region between IR2 and IR3 was designated R3. The two genes, *acf* and orf27 (putatively encoding a type I restriction endonuclease), adjacent to IR2 had 96.8% identities to the counterparts of a SCC element on the chromosome of *S. haemolyticus* JCSC1435. At the other end of R3, there was a second copy of the *ars* operon, which was closest to those on a few *S. aureus* plasmids, e.g. pI258 (GenBank accession no. GQ900378) and pK59 (GenBank accession no. GQ900488) with 92.0% identity and had only 86.4% identity with the first *ars* operon in R2 of WCH1. The intervening genetic components in R3 had lower than 80% identity with the closest matches identified by BLAST and were absent from the chromosome of *S. haemolyticus* JCSC1435. All above findings suggest that all genetic components in R3 had origins other than *S. haemolyticus*. Of note, there were two copies of IS*431* and an IS*Sha1* in R3 but no DR could be identified, suggesting that these MGE were likely to have resulted from homologous recombination rather than direct insertion.

Of note, R3 contained several possible virulence factors. A putative proline permease-encoding *putP* gene was present on R3 and had 78% identity with that of *Staphylococcus saprophyticus* strain ATCC 15305 [23]. *putP* has been identified as a virulence factor in *S. aureus*, contributing to invasive infection [24]. R3 also contained a *feoB*-like gene that was 68% identical to the counterpart of *Staphylococcus carnosus* strain TM300 (GenBank accession no. AM295250). *feoB* has been known as a virulence factor in Gram-negative bacteria, while its virulence status in Gram-positive remains controversial since it has been found conferring virulence in *Streptococcus suis*[25] but not in *Listeria monocytogenesis*[26] and there is no study of *feoB* for staphylococci. In addition, orf32 encodes a putative ABC-type bacteriocin transporter, which might involve in the regulation of virulence factor expression.

In addition, a number of genes encoding products for metabolism, transporting nutrients or detoxifying harmful substances were present in this large region carrying *mecA* (Table 1). The presence of these features could enhance the adaptation of the host strain to variable environment and therefore provided advantages in fitness. Of note, it has been reported that staphylococci are resistant to chromates [27]. A putative chromate transporter gene mediating resistance to chromates was found but with no significant matches in staphylococci. To our knowledge, it is the first time to identify a chromate transporter gene in staphylococci. It also suggests that additional mechanisms are responsible for the chromate resistance in staphylococci.

Although the genetic context of *mecA* was characterized in detail, the exact reason for the absence of *ccr* genes in WCH1 remains undetermined. It is possible that *mecA* was originally carried by a SCC*mec* element with *ccr* genes and the subsequent insertion of an additional IS*431* upstream of *mecA* could give rise to the potential composite transposon, Tn*6191*, together with the already-existed IS*431* downstream of *mecA*. Tn*6191* might have mobilized *mecA* into a new genomic location or alternatively, *ccr* genes could have been deleted due to homologous recombination between multiple copies of IS*431* that were present in WCH1.

## Conclusions

*mecA* was identified in a 40-kb region that contained IR of SCC elements but no *ccr* genes. This large region was very complex in structure and contained multiple genetic components with different origins. Genetic components with various origins were likely introduced in tandem by SCC elements and insertion sequences through insertion and homologous recombination. Two copies of IS*431* bracketed *mecA* and were flanked the characteristic 8-bp direct repeat sequence, suggesting that the two IS*431* might have form a composite transposon with the potential to be active. The IS*431*-formed composite transposon might represent a new mechanism for the mobilization of *mecA* independent of the action of SCC*mec*. The present study aimed to illustrating the complex context of *mecA* but further experiments to demonstrate the activity of Tn*6191* in transposing *mecA* are warranted to confirm the proposed new mechanisms for the mobilization of *mecA*.

## Methods

### Strain and SCC*mec* typing

Clinical isolate WCH1 was recovered from blood collected in West China Hospital, Chengdu, western China, and was obtained as part of standard care. WCH1 was identified as *S. haemolyticus* by partially sequencing the 16S rRNA gene amplified with the universal primers 27 F and 1492R [28]. WCH1 could grow on plates containing 4 μg/ml cefoxitin (Sigma, St Louis, MO). The MIC of cefoxitin against WCH1 was determined using the broth dilution method following the Clinical and Laboratory Standards Institute guidelines [29]. The *mecA* gene and its regulatory genes *mecI* and *mecR1* were detected by PCR [30]. The SCC*mec* typing was carried out using multiplex PCR [30]. The presence of *ccr* genes were also examined by PCR using multiple universal primers as described previously [9].

### PCR mapping and inverse PCR

Two overlapping long-range PCR (Fermentas, Burlington, ON, Canada) were used to obtain two regions, one between *mecA* and orfX and the other between *mecA* and an inverted repeat (IR) sequence of SCC (Figure 1). A few inverse PCR reactions were employed to identify the genetic context of *mecA* with pairs of outwards-facing primers (Table 2 and Figure 1). Genomic DNA of WCH1 prepared using a commercial kit (Tiangen, Beijing, China) was restricted with a certain restriction enzyme (Figure 1), self-ligated with T4 DNA ligase (New England Biolabs, Ipswich, NY) and then used as a template for inverse PCR. The links between genetic elements were confirmed by overlapping long-range PCR (Figure 1, primers listed Table 2).

**Table 2:** Primers used for PCR

**Primer**	**Sequence (5**^′^**-3**^′^)^***a***^	**Target/location**^***b***^	**Reference**
acf-R1	TCCTCAGCATCCTTTTCTTCA	*acf*	This study
arsR-up1	TGTGGATCTATGGAATTGAGGA	upstream of *arsR*	This study
feoB-F1	ATGGTCTCCAAAAAGCATGA	*feoB*	This study
feoB-F2	AAGACAGGAACAAGCGAAACA	*feoB*	This study
feoB-R1	TGGGGCATTGATTACACTGA	*feoB*	This study
IRL-scc	TATCRGWTRATGATGMGGTTT	IRL of SCC*mec*	[7]
IS2	TGAGGTTATTCAGATATTTCGATGT	IS*431*	[31]
MecA147-F	GTGAAGATATACCAAGTGATT	*mecA*	[30]
MecA147-R	ATGCGCTATAGATTGAAAGGA	*mecA*	[30]
orf_1-F1	GAAATGAGCTGAAAGCACGA	*lip*	This study
Orf2_1-R1	TTGGAGGTTTCTCCCCATC	orf24, a Putative multicopper oxidases gene	This study
Orf24-1	CCAAATGAATTGTTAGACGTTG	spacer between *putP* and IS*431*Δ	This study
orfX-F1	GAAAAAGCACCWGAAAMTATGAG	orfX	[7]
SH0128-R1	TTTTGTGGTTGTGACGGTGT	orf45, locus SH0128 in AP006716	This study
ZZ-3	GTGAGGTTGGTGGTGATAAAA	spacer between *putP* and IS*431*Δ	This study
ZZ-4	GCGGGTCCTTCTGGTATAGG	*FAD*	This study
ZZ-11	TATCTCGGGAAATCGATAAAAA	spacer between IS*431* and orf32	This study
ZZ-12	GTTGAAAGGAAACAAAAACTACG	spacer between IS*431* and orf32	This study
ZZ-16	CCGATAACGTCATTCCATCT	orf32, ABC-type bacteriocin transporter gene	This study
ZZ-24	AGCACGACAACAAAAGCATC	orf33	This study
ZZ-28	TGGAGGAGGAGTTTTGGCTA	orf35, chromate transporter	This study
ZZ-29	TACGACATGACCACCTCCAA	orf35, chromate transporter	This study
ZZ-30	GTAGCTGTTGCCATTGTTGC	orf35, chromate transporter	This study
ZZ-31	GCTTGCAGGTCCAGGTAAAA	orf35, chromate transporter	This study
ZZ-32	TGGACGTATCGCTTCAAATG	orf33	This study

^*a*^ D: A, G or T; H: A, C or T: M: A or C; R: A or G; W: A or T; Y: C or T; V: A, C or G.

^*b*^ More information about orfs listed here is available in Table 1.

### Sequencing

Amplicons were sequenced by primer walking using an ABI 3730xl DNA Analyzer (Applied Biosystems, Foster City, CA) at the Beijing Genomics Institute (Beijing, China). Sequences were assembled using the SeqMan II program in the Lasergene package (DNASTAR Inc, Madison, WI) and similarity searches were carried out using BLAST programs (http://www.ncbi.nlm.nih.gov/BLAST/). The putative function of proteins was analyzed using the InterProScan tool (http://www.ebi.ac.uk/Tools/pfa/iprscan/).

**Nucleotide sequences accession number.** The complete sequence of the genetic context of *mecA* in WCH1 has been deposited in GenBank as JQ764731.
